# Blood Cell Intracellular pH Dynamics and Metabolic Biomarkers Under Simulated Postmortem Interval Conditions: An Exploratory Pilot In Vitro Study

**DOI:** 10.3390/ijms27219931

**Published:** 2026-11-06

**Authors:** Paulino D. Ndunduma, Carolina Cordeiro, Susana Faria, M. Begoña Criado, Susana Coimbra, M. Céu Monteiro

**Affiliations:** 1 UCIBIO, Toxicologic Pathology Research Laboratory, CESPU University, 4585-116 Gandra, Portugal; a40311@alunos.cespu.pt (P.D.N.); a41973@alunos.cespu.pt (C.C.); mceu.monteiro@ipsn.cespu.pt (M.C.M.); 2 Associate Laboratory i4HB—Institute for Health and Bioeconomy, CESPU University, 4585-116 Gandra, Portugal; 3 Instituto Superior Politécnico de Benguela, Benguela, Angola; 4 Centre of Mathematics (CMAT), Department of Mathematics, School of Sciences, University of Minho, Campus de Gualtar, 4710-057 Braga, Portugal; sfaria@math.uminho.pt; 5 UCIBIO i4HB, Faculdade de Farmácia, Universidade do Porto, Rua Jorge Viterbo Ferreira 228, 4050-313 Porto, Portugal; 6 UCIBIO, Translational Toxicology Research Laboratory, CESPU University, Avenida Central de Gandra 1317, 4585-116 Gandra, Portugal

**Keywords:** postmortem interval, intracellular pH, flow cytometry, platelets, lactate, ammonia, glucose, thanatochemistry

## Abstract

Intracellular pH (pHi) may provide complementary information on postmortem cellular metabolism and homeostasis, but its temporal behavior remains poorly understood. This pilot in vitro study evaluated temporal changes in platelet pHi and pHi in a mixed non-platelet blood-cell population (predominantly erythrocytes and leukocytes), together with plasma glucose, lactate, and ammonia during a simulated postmortem interval (PMI). Venous blood from eight healthy volunteers was maintained at 24 ± 3 °C and analyzed at 0, 3, 6, and 24 h. Intracellular pH was assessed by BCECF-AM flow cytometry, and metabolites were quantified enzymatically. Platelet and non-platelet-cell pHi remained stable during the first 3 h, decreased significantly at 6 h, and increased toward baseline values at 24 h. Glucose concentrations decreased over time, lactate increased progressively, and ammonia increased significantly only at 24 h. Lactate was associated with pHi in both blood-cell populations. Intracellular pH exhibited a biphasic temporal pattern and distinct relationships with metabolic biomarkers during the simulated PMI. An exploratory mixed-effects model incorporating non-platelet-cell pHi, lactate, and glucose explained a substantial proportion of temporal variability (marginal R^2^ = 0.907) and yielded a leave-one-donor-out cross-validation RMSE of 0.4998 on the log-transformed time scale. These findings support further investigation of multi-marker approaches for PMI estimation.

## 1. Introduction

Accurate estimation of the postmortem interval (PMI) remains a major challenge in forensic science because it can assist in death investigation, event reconstruction and the evaluation of witness statements and alibis [1,2]. Traditional approaches including algor mortis, rigor mortis, livor mortis, decomposition assessment, and forensic entomology, remain widely used but may be influenced by numerous intrinsic and extrinsic factors, resulting in uncertainty in PMI estimates [2,3,4,5].

Consequently, increasing attention has been directed toward molecular and biochemical approaches that reflect the progressive physiological changes occurring after death. Recent advances in PMI estimation have included transcriptomic, proteomic, metabolomic and microbiome-based approaches, as well as machine learning models integrating multiple biological variables. These emerging methodologies may improve predictive accuracy compared with traditional morphology-based assessments, although their forensic implementation remains challenging due to biological and environmental variability [6,7]. No single biomarker has demonstrated sufficient reliability to provide a universally applicable estimate of the PMI, as postmortem changes are influenced by complex interactions between biological and environmental factors [2,8,9,10,11]. Thus, increasing emphasis has been placed on multi-marker strategies that combine complementary parameters to improve PMI estimation [10,11]. Among the different classes of biomarkers investigated, metabolic markers have attracted particular interest because they reflect postmortem alterations in cellular metabolism and homeostasis [10,12,13,14].

Following death, cellular metabolism does not cease immediately but undergoes a progressive transition as oxygen availability declines, energy reserves become depleted, and normal homeostatic mechanisms deteriorate [13,15,16]. Although the cessation of circulation and respiration rapidly disrupts physiological homeostasis, residual cellular metabolic activity and biochemical reactions may persist for a variable period before progressively declining as cellular integrity is lost [11,12,13]. These postmortem processes result in measurable alterations in metabolic activity and intracellular homeostasis, including changes in the concentrations of key metabolites and intracellular acid–base balance [13,15]. Accordingly, metabolic biomarkers such as lactate, ammonia, and glucose have been investigated as potential indicators of postmortem biochemical changes and candidate markers for PMI estimation [12,13,14,15].

Intracellular pH (pHi) may provide complementary information, as alterations in cellular metabolism, energy production, and the accumulation of acidic metabolites following death can affect the intracellular acid–base environment [13,15,17]. The regulation of pHi is closely linked to cellular metabolism and is increasingly recognized as an important determinant of immune-cell function and homeostasis [17]. The assessment of pHi in different blood-cell populations may therefore provide additional insight into the biochemical progression of the postmortem period and its potential relationship with the PMI.

Platelets are highly metabolically active cells and are sensitive to changes in their microenvironment. pHi is closely linked to platelet metabolism and function, including platelet activation, calcium signaling, granule secretion, cytoskeletal rearrangement, and thrombus formation [18]. Platelets possess active mechanisms, including Na^+^/H^+^ exchange, that contribute to the regulation of intracellular acid–base balance [19]. Consequently, changes in platelet pHi may reflect alterations in platelet metabolic and cellular homeostasis and could provide information complementary to that obtained from extracellular biochemical markers. The ability of platelets to retain metabolic and functional properties for several days under optimized storage conditions [20,21] further suggests that measurable intracellular properties may persist beyond the cessation of circulation. Although platelet metabolism and pHi regulation have been extensively investigated in the context of platelet activation and blood-bank storage [18,19,20,21], previous studies have reported changes in platelet count during the early PMI [22]. However, studies specifically assessing temporal changes in platelet pHi under conditions intended to simulate postmortem metabolic deterioration remain scarce. Thus, the extent to which platelet pHi reflects PMI-associated metabolic alterations remains unclear.

Erythrocytes and leukocytes may provide relevant information on postmortem cellular changes. Erythrocytes rely predominantly on anaerobic glycolysis for energy production and may therefore undergo metabolic and acid–base alterations as glucose is consumed and lactate accumulates under oxygen-limited conditions [23,24]. Leukocytes, in contrast, retain more complex metabolic and regulatory functions and may undergo progressive functional and structural deterioration during storage [25]. Leukocytes remain metabolically active after collection and continue to consume energy substrates while maintaining membrane transport processes and pHi regulation [26,27,28,29]. Progressive energy depletion during storage may therefore impair acid–base homeostasis and contribute to pHi alterations, cellular dysfunction, and structural deterioration [17,26,30]. Changes in the intracellular acid–base environment of these blood-cell populations may therefore provide additional information on cellular metabolism and homeostasis during the simulated PMI.

In this exploratory pilot in vitro study, we investigated temporal changes in platelet pHi, the pHi of the non-platelet blood-cell population (predominantly composed of erythrocytes and leukocytes), and the metabolic biomarkers lactate, ammonia, and glucose during storage conditions designed to simulate selected aspects of the postmortem period. The primary objective was to characterize the temporal behavior of these parameters over time. Secondary exploratory objectives were (i) to examine potential associations between pHi measurements and metabolic biomarkers and (ii) to evaluate whether combinations of these variables could improve estimation of elapsed time since blood collection under controlled experimental conditions.

Because the study was performed using blood obtained from living volunteers, the findings should be interpreted as reflecting a simplified experimental model of selected postmortem biochemical processes rather than direct evidence of PMI estimation in forensic casework.

## 2. Results

**Figure 1:**
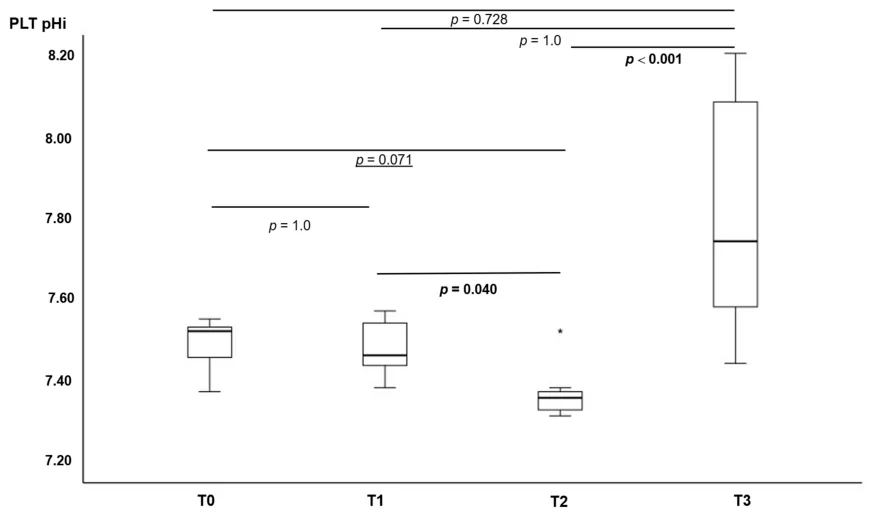
Platelet (PLT) intracellular pH (pHi) at baseline (0 h; T0) and after 3 h (T1), 6 h (T2), and 24 h (T3) of the simulated postmortem interval. Data are presented as median [interquartile range] (boxes) and minimum–maximum values (whiskers). The asterisk (*) marks an outlier.

Regarding platelet pHi (Figure 1 and Table S1), no significant difference was observed between baseline (0 h) and 3 h, although a decreasing trend was evident at 6 h. Platelet pHi decreased significantly between 3 and 6 h, followed by a significant increase between 6 and 24 h. No significant differences were observed between 0 and 24 h or between 3 and 24 h.

**Figure 2:**
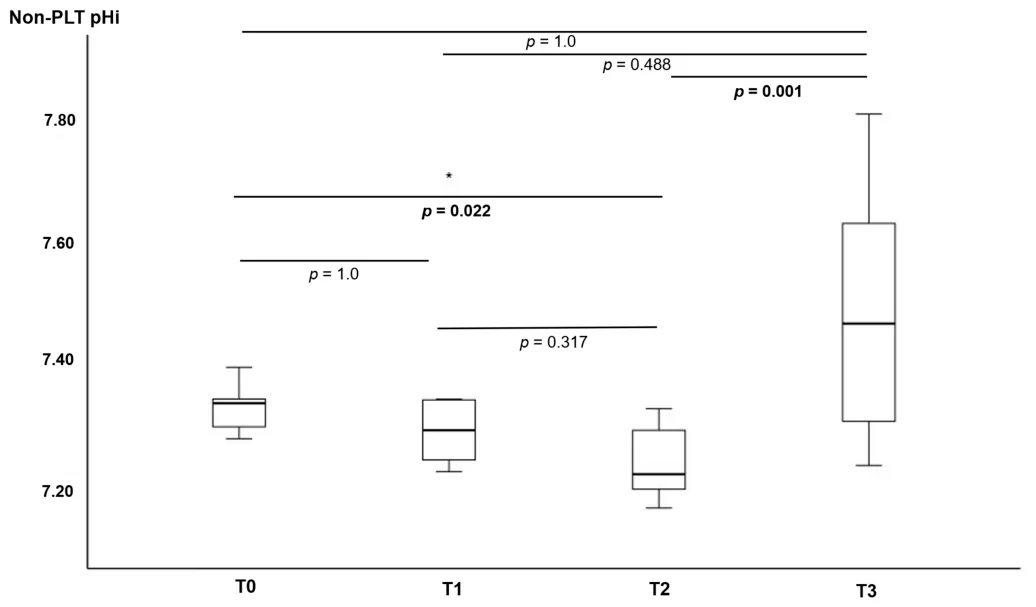
Intracellular pH (pHi) of the non-platelet (non-PLT) blood-cell population at baseline (0 h; T0) and after 3 h (T1), 6 h (T2), and 24 h (T3) of the simulated postmortem interval. Data are presented as median [interquartile range] (boxes) and minimum–maximum values (whiskers). The asterisk (*) marks an outlier.

The flow cytometry method also enabled assessment of pHi in the non-platelet blood-cell population (Figure 2 and Table S1). In this case, pHi did not differ significantly between 0 and 3 h or between 3 and 6 h. However, a significant decrease was observed between 0 and 6 h, followed by a significant increase between 6 and 24 h. No significant differences were detected between 0 and 24 h or between 3 and 24 h.

**Figure 3:**
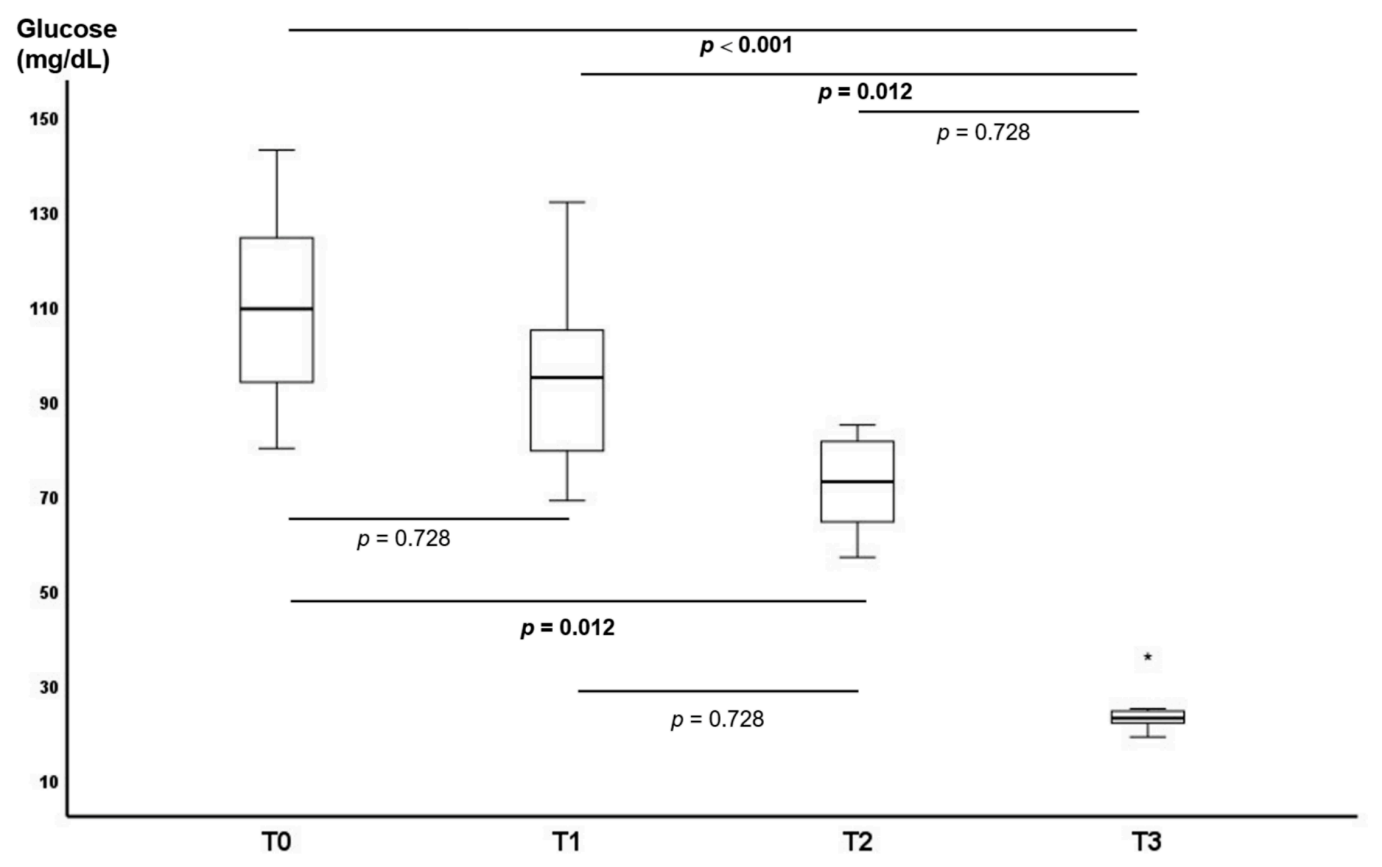
Plasma glucose concentrations at baseline (0 h; T0) and after 3 h (T1), 6 h (T2), and 24 h (T3) of the simulated postmortem interval. Data are presented as median [interquartile range] (boxes) and minimum–maximum values (whiskers). The asterisk (*) marks an outlier.

Glucose concentrations (Figure 3 and Table S1) did not differ significantly between baseline and 3 h, but decreased significantly at 6 h and remained significantly lower at 24 h. No significant difference was observed between 3 and 6 h, whereas glucose concentrations were significantly lower at 24 h than at 3 h. There was also no significant difference between 6 and 24 h.

**Figure 4:**
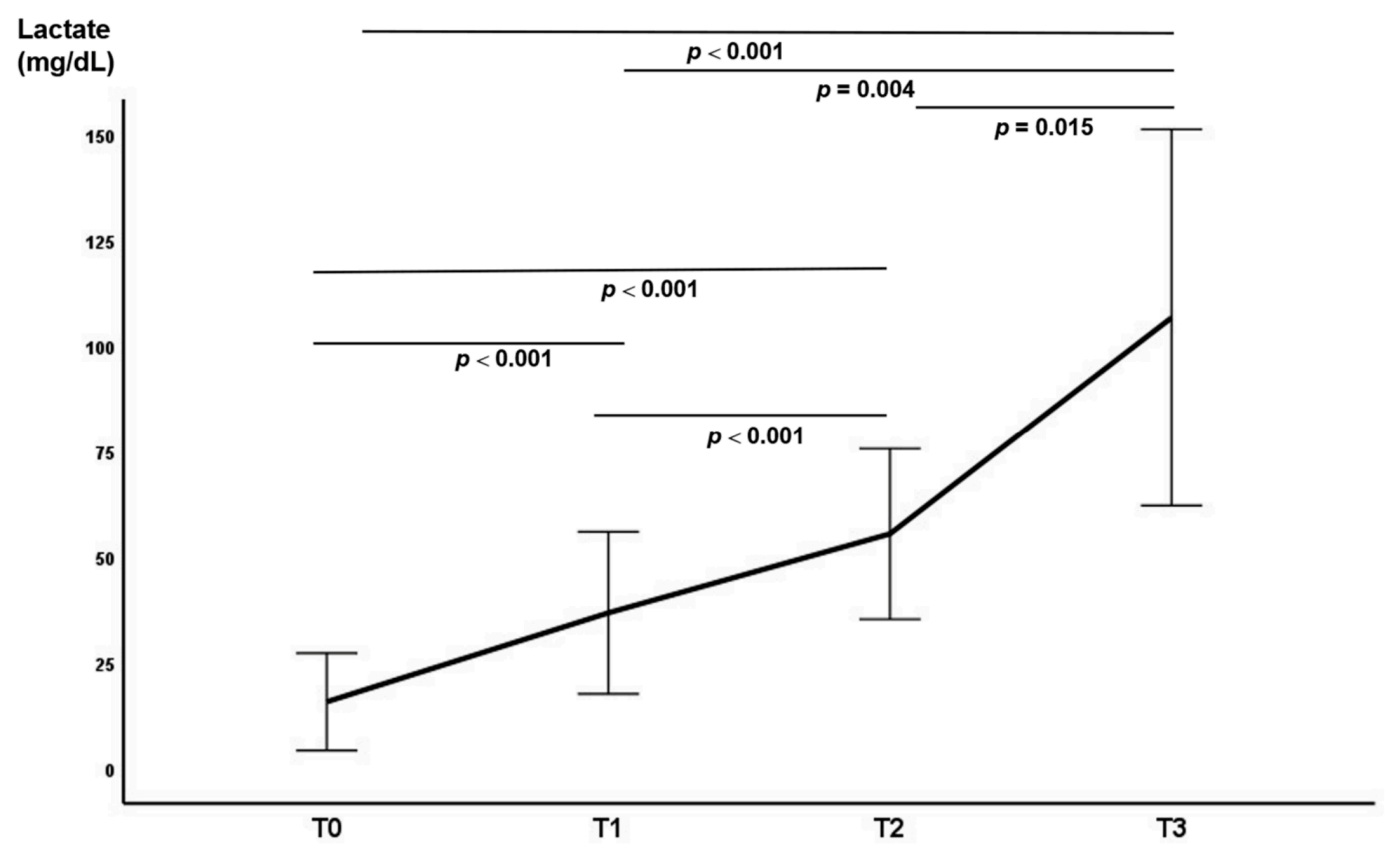
Plasma lactate concentrations at baseline (0 h; T0) and after 3 h (T1), 6 h (T2), and 24 h (T3) of the simulated postmortem interval. Data are presented as mean ± standard deviation.

Lactate concentrations (Figure 4 and Table S1) differed significantly across all time points, showing a progressive increase from baseline to 24 h simulated PMI.

**Figure 5:**
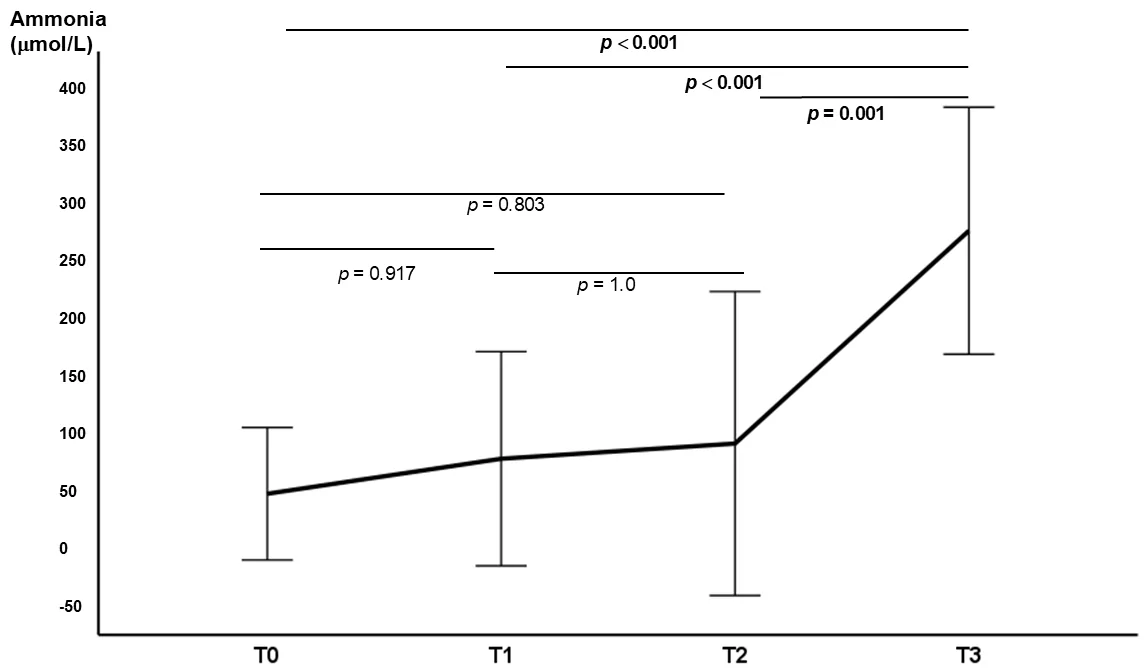
Plasma ammonia concentrations at baseline (0 h; T0) and after 3 h (T1), 6 h (T2), and 24 h (T3) of the simulated postmortem interval. Data are presented as mean ± standard deviation.

Plasma ammonia concentrations (Figure 5 and Table S1) did not differ significantly between baseline and 3 h, baseline and 6 h, or between 3 h and 6 h of the simulated PMI. However, plasma ammonia concentrations were significantly higher at 24 h than at baseline, 3 h, and 6 h.

Linear mixed-effects modelling (Table 1) showed that, in the model including lactate, both time (F(3,17.707) = 12.417, *p* = 0.002) and lactate (β = 0.006, 95% CI: 0.002–0.011, *p* = 0.009) were significantly associated with platelet pHi. In the model including ammonia, ammonia was significantly associated with platelet pHi (β = 0.001, 95% CI: 0.000–0.002, *p* = 0.032), whereas the effect of time almost reached statistical significance (F(3,21.036) = 2.983, *p* = 0.054). In the model including glucose, time remained significantly associated with platelet pHi (F(3,19.449) = 5.439, *p* = 0.007), whereas glucose was not significantly associated with platelet pHi (β = −0.001, 95% CI: −0.005 to 0.003, *p* = 0.657).

For the non-platelet blood-cell population (Table 1), both time (F(3,16.298) = 5.200, *p* = 0.010) and lactate (β = 0.006, 95% CI: 0.002–0.010, *p* = 0.006) were significantly associated with pHi in the model including lactate. In contrast, neither ammonia (β = 0.000, 95% CI: −0.001 to 0.001, *p* = 0.755) nor glucose (β = −0.002, 95% CI: −0.005 to 0.002, *p* = 0.295) was significantly associated with pHi, and the effect of time was not significant in these models (*p* = 0.121 and *p* = 0.094, respectively).

In a complementary analysis, a mixed-effects regression model was developed to estimate elapsed sampling time from the measured biomarkers, using log(time + 1) as the response variable, where log denotes the natural logarithm. The response variable was log-transformed using log(time + 1) to reduce the influence of larger sampling-time values and potentially improve the validity of the model assumptions. A mixed-effects modelling framework was used to account for the dependence between repeated measurements obtained from the same volunteer. After exploratory backward elimination from the initial set of five candidate predictors, the final model retained the non-platelet blood cells pHi, lactate, and glucose (Table 2); whereas platelet pHi and ammonia were excluded.

The final model achieved a marginal R^2^ of 0.907, with a residual standard deviation (SD) of 0.3514. Using leave-one-donor-out cross-validation, with backward variable selection repeated within each training set, the model yielded an root mean square error (RMSE) of 0.4998 on the log(time + 1) scale. After back-transformation of the individual cross-validated predictions to the original time scale, the corresponding RMSE was 6.05 h. The estimated donor-specific random-intercept SD was approximately 0.0001, suggesting a near-zero variance component under the current model specification.

The final mixed-effects model is expressed as

$$\log\left({time}_{ij}+1\right)=13.1453-1.6054\times {nPLT.pHi}_{ij}+0.022805\times {lactate}_{ij}-0.012437\times {glucose}_{ij}+b_{i}+\varepsilon_{ij}$$

where time*_ij_* denotes the elapsed time since blood collection, in hours, for observation *j* from donor *i*; nPLT.pHi denotes the pHi of the non-platelet blood-cell population; and lactate and glucose are expressed in mg/dL. The term b*_i_* represents the donor-specific random intercept, and ε*_ij_* denotes the residual error for observation *j* from donor *i*.

The corresponding point estimate of elapsed time since blood collection, in hours, is obtained by inverse transformation:

$$\hat{time}=\exp\left(13.1453-1.6054\times nPLT.pHi+0.022805\times lactate-0.012437\times glucose\right)-1.$$

Non-platelet blood-cell population pHi was negatively associated with transformed elapsed time (β = −1.6054, *p* = 0.0037), whereas lactate was positively associated (β = 0.022805, *p* < 0.001) and glucose negatively associated (β = −0.012437, *p* = 0.0005) (Table 2). These are adjusted associations and do not demonstrate independent causal effects.

**Figure 6:**
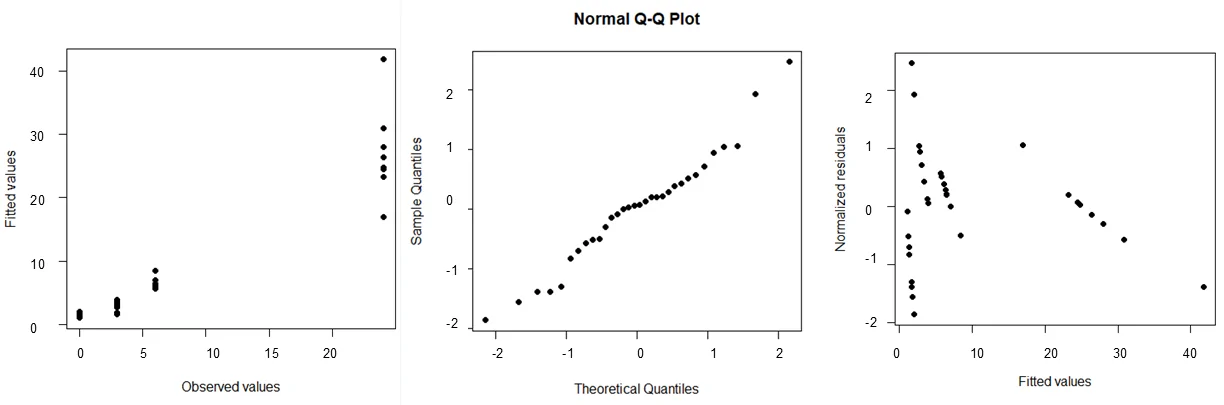
Exploratory diagnostic plots for the final prediction model: (from left to right) observed versus fitted elapsed time, normal Q–Q plot of residuals, and standardized residuals versus fitted values.

The normal Q–Q plot for the final model showed an approximately linear central pattern, with some departures at the extremes (Figure 6). The Shapiro–Wilk test did not indicate a statistically significant deviation from normality (W = 0.9691, *p* = 0.4743).

The residual-versus-fitted and observed-versus-fitted plots were examined to assess potential systematic patterns, deviations from the assumed model structure, and evidence of non-constant residual variance. The observed-versus-fitted plot indicated moderate agreement between observed and fitted values. While most data points were well approximated by the model, one higher observed value exerted moderate influence on the fitted results. The normal Q-Q plot showed minor departures in the tails; however, formal normality tests confirmed that the normality assumption was not violated (*p* = 0.4743). The residual-versus-fitted plot revealed some clustering and mild non-random patterns, suggesting moderate heteroscedasticity. Despite this, the model is considered to provide a reasonable fit to the data.

## 3. Discussion

To the best of our knowledge, this is the first exploratory pilot in vitro study to investigate platelet pHi and pHi in a non-platelet blood-cell population, alongside the metabolic biomarkers lactate, ammonia, and glucose, in the context of simulated PMI. Rather than validating individual biomarkers for PMI estimation, this study aimed to explore their temporal behavior and their potential utility, individually and in combination, as candidate parameters for inclusion in a multi-marker panel for PMI estimation. Although blood pH has been previously assessed as a postmortem marker [13], pHi may offer additional insights into blood cell metabolism and the cellular and biochemical changes occurring under simulated postmortem conditions. According to our findings, platelet pHi and non-platelet pHi (erythrocyte and leukocyte cell population) remained relatively stable during the first 3 h, followed by a significant decrease at 6 h and an increase toward baseline values at 24 h.

In human blood and rat blood stored in tubes, blood pH was reported to decrease slightly during the first 24 h of the simulated PMI, and the same trend was observed up to 96 h [13]. Whereas blood pH reflects changes in the extracellular environment resulting from the accumulation of metabolites released by blood cells, pHi provides a more direct measure of the intracellular acid–base state and is influenced by cellular metabolic activity, buffering capacity, and membrane transport processes. Therefore, pHi may provide complementary information on postmortem cellular alterations that cannot be fully captured by measurements of extracellular blood pH alone. Consistent with previous in vitro blood-storage studies, glucose decreased whereas lactate and ammonia increased over time [13]. However, the biphasic behavior observed for pHi has not, to our knowledge, been previously reported, highlighting the need for further investigation and independent validation.

From 0 to 3 h of the simulated PMI, the absence of significant changes in blood-cell pHi suggests that intracellular acid–base homeostasis remains relatively preserved during this initial period. In contrast, the significant decrease in blood-cell pHi observed at 6 h may reflect a progressive shift toward anaerobic glycolysis under simulated postmortem conditions, as oxygen availability progressively becomes limited under the storage conditions and blood cells rely increasingly on residual glycolytic metabolism. These changes may promote intracellular acidification through the accumulation of metabolic products and progressive disruption of proton-regulating mechanisms. Blood stored under simulated postmortem conditions has been reported to undergo progressive biochemical alterations, including changes in pH and blood-cell membrane properties. The decrease in blood pH has been associated with the accumulation of metabolites and ions, such as lactate and H^+^, resulting from progressive blood-cell deterioration [13]. Thus, the decrease in pHi observed at 6 h suggests that residual glycolytic activity in blood cells persists during this initial period, while the mechanisms responsible for maintaining pHi progressively deteriorate.

Interestingly, by 24 h, pHi had increased significantly compared with 6 h and returned to values that were no longer significantly different from baseline. This apparent normalization is unlikely to represent recovery of normal cellular function. Several mechanisms could potentially contribute to this pattern, including progressive alterations in cellular integrity, disruption of transmembrane proton gradients, changes in intracellular ion distribution, and shifts in the composition of the surviving measurable cell population. Another possible explanation is that progressive metabolic exhaustion may reduce intracellular acid production at later time points. However, these mechanisms were not directly evaluated in the present study. Therefore, the observed increase in pHi should be interpreted cautiously as a descriptive finding whose biological basis remains to be elucidated. Regarding metabolic markers, plasma glucose concentrations did not differ significantly between 0 and 3 h but decreased significantly at both 6 h and 24 h compared with baseline. In contrast, plasma lactate concentrations increased progressively throughout the simulated PMI. Previous studies have also investigated circulating glucose and lactate levels as potential biochemical markers of PMI [12,13,14,31]. Consistent with our findings, lactate concentrations have been reported to increase with increasing PMI in human cadavers [32], whereas decreases in glucose and increases in lactate have also been described in vitreous humor during the postmortem period [33]. Although findings have varied according to biological matrix, study design, and postmortem conditions, these observations collectively support the occurrence of progressive postmortem metabolic alterations. Together, these metabolic markers may provide relevant information on postmortem biochemical changes and therefore warrant further investigation as components of a multi-marker approach to PMI estimation. At 6 h, the combination of decreased glucose, increased lactate, and reduced blood-cell pHi is consistent with ongoing glycolytic metabolism under simulated PMI conditions. The temporal coexistence of these changes suggests that glycolysis may contribute to intracellular acidification during the early stages of simulated PMI, even though the underlying mechanisms were not directly assessed.

Although lactate continued to accumulate at 24 h, blood-cell pHi returned to values that were not significantly different from baseline. This apparent dissociation suggests that pHi was no longer determined solely by anaerobic glycolysis but was increasingly influenced by progressive blood-cell deterioration and disruption of cellular homeostasis. The significant increase in plasma ammonia at 24 h is consistent with the progressive degradation of blood-cell constituents under simulated PMI conditions. An increase in circulating ammonia concentrations over the 0–96 h period was also reported in rat and human blood stored in vitro [13]. Ammonia exists in equilibrium between NH_3_ and NH_4_^+^; because NH_3_ is membrane permeable, it can diffuse across cell membranes and bind protons to form NH_4_^+^, potentially contributing to intracellular alkalinization. Although ammonia accumulation coincided temporally with the increase in pHi, the present data do not permit any inference regarding causality. Therefore, the possibility that ammonia contributes to intracellular alkalinization remains speculative and requires direct experimental investigation.

The mixed-effects analyses provided further insight into these temporal patterns. These analyses supported a significant temporal effect on both platelet pHi and non-platelet blood-cell population pHi, indicating that pHi varied throughout the simulated PMI period. Lactate was the only metabolic parameter consistently associated with pHi in both platelet and non-platelet blood cells populations, suggesting that changes in extracellular lactate were related to alterations in pHi in these cell populations. However, the interpretation of this association requires caution because lactate concentrations increased progressively throughout the experimental period, whereas pHi exhibited a biphasic pattern. This discrepancy suggests that lactate alone is unlikely to explain the observed pHi changes and that additional processes may contribute to pHi regulation during simulated PMI. In contrast, glucose was not significantly associated with pHi, suggesting that glucose concentration alone may not adequately reflect the intracellular metabolic changes occurring during the simulated PMI. Ammonia showed a significant association only with platelet pHi. Given the exploratory nature of the study, this finding should be interpreted with caution, although it may indicate differences in the metabolic responses of distinct blood-cell populations. The different patterns observed for platelets and the non-platelet population may reflect differences in cellular metabolism and acid–base regulation, as well as differences in susceptibility to progressive deterioration under simulated PMI conditions. Collectively, these findings indicate that pHi changes follow a non-linear temporal pattern during simulated PMI and are likely influenced by multiple cellular and metabolic processes.

The exploratory prediction model addressed a different objective from the mixed-effects analyses. Whereas the latter examined associations between blood-cell pHi and individual metabolic parameters, the prediction model evaluated whether a combination of biomarkers could estimate elapsed time since blood collection. In this context, biomarkers not significantly associated with pHi individually, such as glucose, may still contribute useful predictive information when considered jointly with other variables.

The final three-predictor model incorporated non-platelet-cell pHi, lactate, and glucose and explained a substantial proportion of the observed variability (marginal R^2^ = 0.907). However, this result should be interpreted cautiously given the small sample size, the exploratory variable-selection strategy, and the absence of external validation. Although leave-one-donor-out cross-validation reduced within-participant information leakage, model performance was assessed only internally and under the specific experimental conditions studied (0–24 h, 24 ± 3 °C). Consequently, the model should be regarded as a hypothesis-generating framework rather than a validated estimator of forensic PMI.

Several limitations should be considered when interpreting these findings. First, the study was conducted using an in vitro blood storage model and therefore does not reproduce the complex physiological, biochemical, and environmental changes occurring after death in the human body. Consequently, the findings should be interpreted as reflecting cellular and biochemical alterations under simulated PMI conditions rather than actual postmortem processes. Second, the small sample size limits statistical power and generalizability, and the findings require confirmation in larger and independent cohorts. Third, measurements were performed at only four time points (0, 3, 6, and 24 h), limiting the characterization of the precise timing of the observed pHi transitions. Future studies should include more frequent sampling, particularly between 6 and 24 h, to provide a more detailed characterization of the temporal trajectories of pHi and related biomarkers and to better define the timing of the observed transitions. Fourth, pHi was assessed in a combined erythrocyte/leukocyte population rather than in individual cell types, precluding evaluation of cell-specific contributions. Fifth, although the observed patterns were consistent with residual glycolytic activity, cellular deterioration, and altered membrane homeostasis, these mechanisms were not directly assessed through measurements of oxygen availability, ATP, membrane integrity, cell viability, or other indicators of cellular damage. Therefore, the proposed mechanistic explanations should be regarded as hypotheses rather than demonstrated pathways. In addition, the distinction between platelet and non-platelet populations was based on light-scatter characteristics rather than specific immunophenotypic markers. Future studies incorporating specific cell-identification markers could improve population discrimination and provide more precise insights into cell-type-specific contributions to the observed alterations. Finally, the observed associations do not establish causality, and the predictive model, developed in a small cohort and evaluated only through internal validation, requires external validation before any forensic application can be considered.

Despite these limitations, the study has several strengths. The longitudinal repeated-measures design allowed the characterization of temporal changes within individuals while minimizing inter-individual variability. In addition, the simultaneous assessment of blood-cell pHi and metabolic biomarkers provided complementary information on cellular and biochemical alterations occurring during simulated PMI and enabled the identification of a two-phase pHi response. These findings provide a basis for future investigations in larger cohorts and authentic postmortem samples.

Overall, this exploratory in vitro study suggests that blood-cell pHi undergoes dynamic changes during simulated PMI and may provide information complementary to that obtained from conventional metabolic biomarkers. The observed temporal patterns and biomarker associations support further investigation of blood-cell pHi as a complementary component of multi-parameter approaches for PMI estimation. However, these findings remain preliminary and require validation in larger studies and authentic postmortem samples before any forensic application can be considered.

## 4. Materials and Methods

### 4.1. Blood Sample Collection and Experimental Design

This study was conducted in accordance with the Declaration of Helsinki, and approved by the Ethics Committee of CESPU University (26/CE-IUCS/2025). Human venous blood was selected as the biological matrix for the assessment of time-dependent changes in blood-cell pHi and selected metabolic biomarkers under controlled in vitro conditions. Blood represents a valuable source of PMI-related biomarkers, as postmortem biochemical processes may result in measurable alterations in cellular and metabolic parameters [14,34].

Eight independent venous blood samples were collected from healthy volunteers after written informed consent was obtained. The inclusion criteria comprised healthy adults with no known acute or chronic disease at the time of blood collection. Of the participants, 75% were female, with a mean age of 43 ± 14 years (range: 23–60 years). As this was an exploratory pilot in vitro study designed to evaluate changes in the concentrations of selected parameters during a simulated PMI under controlled laboratory conditions, a convenience sample of eight healthy blood donors was used. The primary objective was to characterize the temporal behavior of these parameters and obtain preliminary estimates of the magnitude and variability of the observed changes, rather than to provide population-level estimates or establish reference values.

Each venous blood sample was collected using a 10.0 mL sterile syringe and immediately distributed into three plastic tubes: one 2.0 mL tube containing 3.2% sodium citrate (blood-to-anticoagulant ratio 9:1) and two 3.5 mL lithium heparin tubes (18 IU heparin per mL of blood; lyophilized anticoagulant); 3.5 mL of blood was transferred into each heparin tube and 2.0 mL into the citrate tube.

Blood samples were maintained in closed tubes throughout the experimental period. pHi and metabolic biomarkers were assessed immediately after blood collection (0 h) and at 3, 6, and 24 h after collection. For plasma biochemical analyses, 1.75 mL of blood was withdrawn at each time point from the heparinized tubes. One heparin tube was used for the 0 h and 3 h samplings, whereas the second heparin tube was used for the 6 h and 24 h samplings. For pHi determination, aliquots were withdrawn directly from the sodium citrate tube at each time point and processed immediately. Throughout the experimental period, blood samples were maintained under controlled laboratory conditions. Temperature was monitored throughout the experimental period and remained at 24 ± 3 °C. Blood samples were stored in closed collection tubes and protected from light until processing. All samples were handled under identical laboratory conditions to minimize preanalytical variability.

At each predefined time point, 1.75 mL of blood was centrifuged at 1500× *g* for 15 min. Plasma was separated immediately after centrifugation, aliquoted, and stored at −80 °C without delay. Samples remained frozen until completion of sample collection and were subsequently analyzed in a single analytical batch. Each aliquot was thawed only once before analysis, and no additional freeze–thaw cycles were performed.

Hemolysis was assessed by macroscopic visual inspection after plasma separation. No evidence of hemolysis was observed in any sample.

### 4.2. pHi and Metabolic Biomarkers Analysis

#### 4.2.1. Blood-Cell pHi

Flow cytometry enables rapid, high-throughput, multiparametric analysis at the cellular level and has shown potential for forensic applications [35]. In this context, flow cytometry-based assessment of blood-cell pHi may represent a promising approach for investigating postmortem cellular changes and identifying candidate biomarkers of the PMI.

Prior to the experimental phase, the protocol for pHi determination was optimized to define sample dilution, incubation, and acquisition conditions that minimize methodological variability. During the study, all samples were processed following the same standardized procedure. Blood samples were subjected to identical dilution and incubation conditions, and flow cytometric acquisition was performed within a standardized time interval after sample collection for all time points. Instrument settings and sample handling procedures were kept constant throughout the study to minimize potential effects of sample preparation on pHi measurements.

For pHi evaluation, the fluorescent probe BCECF-AM (2′,7′-bis(2-carboxyethyl)-5,6-carboxyfluorescein acetoxymethyl ester) was used [36]. This pH-sensitive probe is a weak acid with a pKa close to 7.0 (approximately 7.5 at room temperature). The deprotonated form of BCECF emits fluorescence at 530 nm (FL1), whereas fluorescence emission at 630 nm (FL3) is pH-independent [37].

The assay was performed using whole blood collected in sodium citrate and diluted in HEPES (4-(2-hydroxyethyl)-1-piperazineethanesulfonic acid) buffer (pH 7.4). Whole-blood samples were diluted 1:10 in Na^+^-HEPES buffer and incubated with BCECF-AM at a final concentration of 4 µM for 15 min at 37 °C. Subsequently, 50 µL aliquots were diluted in 950 µL of Na^+^-HEPES buffer (137 mM NaCl, 3.3 mM NaH_2_PO_4_, 5 mM D-glucose, 2.7 mM KCl, 3.8 mM HEPES, and 0.98 mM MgCl_2_; pH 7.4).

To quantify pHi, a calibration curve was generated by resuspending dye-loaded cells in high-K^+^ HEPES buffer (140 mM KCl) containing the proton ionophore nigericin (2 uM) [38]. For each donor a calibration curve was established by diluting BCECF-AM-loaded whole-blood samples in K^+^-HEPES buffer adjusted to pH values ranging from 6.0 to 8.0 (6.0; 6.5; 7.0; 7.5; 8.0); BCECF-AM-loaded blood cells were incubated in the corresponding calibration buffers for 5 min at room temperature to promote equilibration between intracellular and extracellular pH. Following incubation, fluorescence intensities were measured in the FL1 (530 nm) and FL3 (630 nm) channels, and the FL1/FL3 ratio was calculated for each calibration condition These fluorescence ratios were plotted against the corresponding buffer pH values to establish donor-specific calibration curves. The resulting calibration curves were used to convert the fluorescence ratios obtained from experimental samples into absolute pHi values. The flow cytometer (BD Accuri™ C6 Plus; BD Biosciences, Franklin Lakes, NJ, USA) was configured to acquire forward scatter (FSC) and side scatter (SSC) signals for the identification of blood-cell populations based on cell size and granularity. An argon laser operating at 488 nm was used to excite BCECF. Fluorescence emissions at 530 nm (FL1) and 630 nm (FL3) were collected using logarithmic amplification, and the FL1/FL3 fluorescence ratio was calculated. Under the calibration conditions employed, an increase in this ratio was interpreted as an increase in pHi.

Ammonium chloride (10 mM) and sodium propionate (100 mM) were used as positive controls for intracellular alkalinization and acidification, respectively. The expected increases and decreases in the FL1/FL3 fluorescence ratio following these treatments confirmed the sensitivity of the method to changes in pHi. This approach also enabled the assessment of pHi in a mixed non-platelet blood-cell population, composed predominantly of erythrocytes and leukocytes. Platelet and non-platelet-cell populations were identified by flow cytometry based on their FSC and SSC characteristics, which reflect cell size and complexity, respectively. Platelets were identified within the region corresponding to smaller particles, whereas the remaining cellular events were classified as the non-platelet population, predominantly comprising erythrocytes and leukocytes (Figure S1). This scatter-based approach allowed the assessment of pHi in two operationally defined blood-cell populations. Accordingly, the non-platelet population was considered a heterogeneous cellular fraction rather than a specific cell subtype. Separate calibration curves were generated for platelet and non-platelet populations to enable the independent determination of pHi in each population. All pHi measurements were performed in duplicate.

#### 4.2.2. Metabolic Markers

Plasma concentrations of lactate and glucose were determined using enzymatic colorimetric methods (Lactate LO-POD and Glucose LQ GOD-POD, respectively; Spinreact, Barcelona, Spain), according to the manufacturer’s instructions. Lactate and glucose measurements were performed in triplicate and duplicate, respectively.

For lactate determination, lactate is oxidized by lactate oxidase to pyruvate and hydrogen peroxide (H_2_O_2_). In the presence of peroxidase, H_2_O_2_ reacts with 4-aminophenazone and 4-chlorophenol to form a colored quinoneimine compound, which was measured photometrically at 505 nm. The intensity of the color formed is proportional to the lactate concentration in the sample.

For glucose determination, glucose oxidase catalyzes the oxidation of glucose to gluconic acid and H_2_O_2_. In the presence of peroxidase, the generated H_2_O_2_ reacts with phenol and 4-aminophenazone to form a colored quinoneimine compound (measured photometrically at 505 nm), the intensity of which is proportional to the glucose concentration in the sample.

Plasma ammonia concentrations were determined using an enzymatic UV method (Spinreact, Barcelona, Spain), according to the manufacturer’s instructions. Measurements were performed in duplicate. In this assay, ammonia reacts with α-ketoglutarate and NADPH in the presence of glutamate dehydrogenase to produce glutamate and NADP^+^. The decrease in absorbance at 340 nm, resulting from NADPH oxidation, is directly proportional to the ammonia concentration in the plasma sample.

Internal quality control was performed using Spintrol “H” Normal and Spintrol “H” Pathological control materials (Spinreact, Barcelona, Spain), according to the manufacturer’s recommendations.

### 4.3. Data Analysis

Statistical analyses were performed using IBM SPSS Statistics for Windows, version 29.0 (IBM Corp., Armonk, NY, USA). The exploratory predictive modelling analysis was conducted in R version 4.6.1 (R Foundation for Statistical Computing, Vienna, Austria).

For each variable, duplicate or triplicate technical measurements were averaged to obtain a single value per donor at each sampling time point. Thus, all statistical analyses were conducted using one biological observation per donor per time point. Technical replicates were used solely to improve analytical precision and were not treated as independent observations. Data normality was assessed using the Shapiro–Wilk test prior to selection of parametric or non-parametric methods.

Differences between time points were evaluated using repeated-measures analysis of variance (ANOVA) for normally distributed data. The assumption of sphericity was assessed using Mauchly’s test and, when violated, appropriate corrections were applied. Post hoc pairwise comparisons were performed using paired *t*-tests with Bonferroni adjustment. For non-normally distributed data, differences between time points were analyzed using the Friedman test, followed by Dunn–Bonferroni-adjusted pairwise comparisons.

To investigate the association between blood cells pHi and metabolic parameters during the simulated PMI, separate linear mixed-effects models were constructed for platelet pHi and for the pHi of the non-platelet blood-cell population. In each model, pHi was specified as the dependent variable, time was included as a fixed categorical effect, and glucose, lactate, or ammonia concentrations were entered individually as fixed continuous covariates. Sample ID was included as a random intercept to account for repeated measurements within samples across the four time points. The significance of fixed effects was assessed using Type III tests. Regression coefficients (β), 95% confidence intervals (95% CIs), and associated *p*-values were reported. The normality assumption for linear mixed-effects models was evaluated by inspection of residual Q-Q plots and histograms.

In a separate exploratory analysis designed to estimate elapsed time since blood collection, a linear mixed-effects model was fitted with the elapsed sampling time as the outcome. Because the distribution of sampling times (0, 3, 6, and 24 h) was markedly right-skewed and preliminary analyses indicated deviations from residual normality, time was transformed as log(time + 1), allowing inclusion of baseline (0 h) measurements. This transformation improved the distributional properties of model residuals. Platelet pHi, non-platelet-cell pHi, lactate, glucose, and ammonia were initially entered as fixed-effect predictors, while donor identity was included as a random intercept to account for repeated measurements. An exploratory backward-elimination procedure removed predictors with weak statistical evidence, leading to a final, more parsimonious model containing non-platelet-cell pHi, lactate, and glucose.

Model fit was described using the marginal coefficient of determination (R^2^), reflecting the contribution of fixed effects, and the residual and random-intercept SD. Predictive performance was assessed using leave-one-donor-out cross-validation: at each iteration, all measurements from one volunteer were withheld, the model coefficients were fitted using the remaining volunteers, and predictions were compared with the withheld observations. The RMSE was calculated on the log(time + 1) scale. Residual diagnostics included visual inspection of a normal Q–Q plot and residuals versus fitted values, together with the Shapiro–Wilk test. Fixed-effect estimates were inverse-transformed as exp(predicted log(time + 1)) − 1 to express illustrative predictions in hours. Given the small number of donors and the exploratory nature of the data-driven variable-selection procedure, results should be interpreted as hypothesis-generating rather than confirmatory.

No outliers were excluded from the statistical analyses. Given the exploratory nature of this preliminary study, the small sample size (*n* = 8), and the use of technical replicates for biochemical measurements (duplicates or triplicates, as appropriate), all observations were retained to preserve biological variability and minimize potential bias associated with the exclusion of individual data points.

Data are presented as mean and SD for normally distributed variables and as median and interquartile range (IQR) for non-normally distributed variables; results are presented as line graphs or boxplots, as appropriate. Statistical significance was set at *p* < 0.05.

## Figures and Tables

**Table 1:** Effect of time and biochemical markers on intracellular pH (pHi) assessed by linear mixed models.

Dependent Variable	Predictor	F (df1, df2)	*p*-Value	β (95% CI)
Platelet pHi	Time Lactate	12.417 (3, 17.707)	0.002	---
		9.470 (1, 13.286)	0.009	0.006 (0.002 to 0.011)
	Time Ammonia	2.983 (3, 21.036)	0.054	---
		5.122 (1, 26.988)	0.032	0.001 (0.000 to 0.002)
	Time Glucose	5.439 (3, 19.449)	0.007	---
		0.205 (1, 14.094)	0.657	−0.001 (−0.005 to 0.003)
Non-platelet blood cell pHi	Time Lactate	5.200 (3, 16.298)	0.010	---
		10.949 (1, 12.001)	0.006	0.006 (0.002 to 0.010)
	Time Ammonia	2.180 (3, 20.763)	0.121	---
		0.100 (1, 20.671)	0.755	0.000 (−0.001 to 0.001)
	Time Glucose	2.466 (3, 18.666)	0.094	---
		1.189 (1, 13.387)	0.295	−0.002 (−0.005 to 0.002)

CI is confidence interval.

**Table 2:** Fixed-effect coefficients of the final mixed-effects model for log(time + 1), with 95% confidence intervals (CI).

Fixed Effect	β	95% CI	*p*-Value
Intercept	13.1453	5.7037 to 20.5868	0.0014
Non-platelet blood cell pHi	−1.6054	−2.6270 to −0.5839	0.0037
Lactate	0.022805	0.016207 to 0.029403	<0.001
Glucose	−0.012437	−0.018713 to −0.006160	0.0005

pHi, intracellular pH.

## Data Availability

The original contributions presented in this study are included in the article. The raw data supporting the conclusions of this article will be made available by the authors upon request. Further inquiries can be directed at the corresponding authors.

## Supplementary Materials

The following supporting information can be downloaded at https://www.mdpi.com/article/10.3390/ijms27219931/s1.

## Abbreviations

The following abbreviations are used in this manuscript:

CI	Confidence interval
FSC	Forward scatter
IQR	Interquartile range
pHi	Intracellular pH
PLT	Platelet
PMI	Postmortem interval
RMSE	Root mean square error
SSC	Side scatter
