# Supplementary material for: Blood Cell Intracellular pH Dynamics and Metabolic Biomarkers Under Simulated Postmortem Interval Conditions: An Exploratory Pilot In Vitro Study
Source: Int J Mol Sci. 2026 Nov 6;27(21):9931. doi: 10.3390/ijms27219931 (PMC13764077; doi:10.3390/ijms27219931)
Supplement: Supplementary file 1 [file ijms-27-09931-s001.zip › ijms-4667973-supplementary.pdf]

Table S1. Platelet intracellular pH (pHi), non-platelet blood cell population pHi, and plasma concentrations of glucose, lactate and ammonia at baseline (0 h; T0) and after 3 h (T1), 6 h (T2), and 24 h (T3) of the simulated postmortem interval.

	T0	T1	T2	T3
Platelet pHi	7.52 [7.44 - 7.53]	7.46 [7.43 - 7.55]	7.36 [7.32 - 7.38] **a, b**	7.75 [7.54 - 8.13] **c**
Non-platelet pHi	7.33 [7.29 - 7.34]	7.29 [7.23 - 7.34]	7.21 [7.19 - 7.30] **d**	7.46 [7.28 - 7.68] **e**
Glucose (mg/dL)	110 [91 - 126]	95 [75 - 108]	73 [62 – 83] **f**	23 [22 – 25] **g, h**
Ammonia (µmol/L)	46.0 ± 28.4	76.0 ± 45.9	89.0 ± 65.1	271.4 ± 52.9 **i, j, k**
Lactate (mg/dL)	15.7 ± 5.7	36.7 ± 9.6 **l**	55.4 ± 10.1 **m, n**	99.2 ± 34.6 **o, p, q**

Data are presented as median [interquartile range] or as mean ± standard deviation.

a *p* vs T0 = 0.071; **b** *p vs* T1 = 0.040; **c** *p vs* T2 < 0.001

d *p* vs T0 = 0.022; **e** *p vs* T2 = 0.001

f *p* vs T0 = 0.012; **g** *p* vs T0 < 0.001; **h** *p* vs T1 = 0.012

i *p* vs T0 < 0.001; **j** *p vs* T1 < 0.001; **k** *p vs* T2 = 0.001

l *p* vs T0 < 0.001; **m** *p* vs T0 < 0.001; **n** *p* vs T1 < 0.001; **o** *p* vs T0 < 0.001; **p** *p* vs T1 = 0.004; **q** *p* vs T2 = 0.015

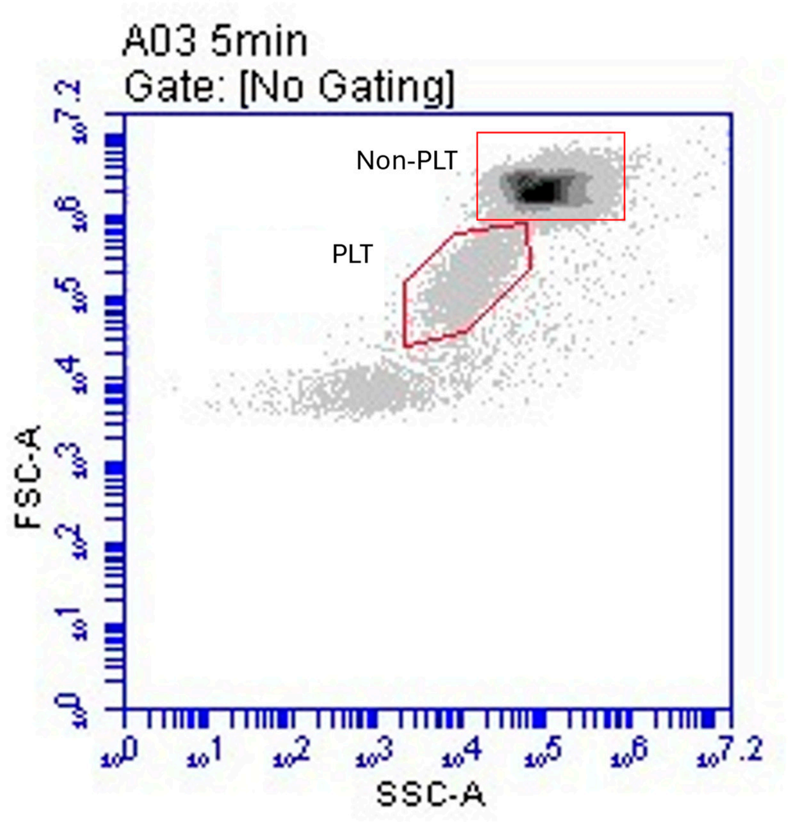

Figure S1. Identification of platelet (PLT) and non-platelet (non-PLT) populations in whole blood based on forward (FSC) and side (SSC) light-scattering properties.
